# Short-Term Cumulative Exposure to Ambient Traffic-Related Black Carbon and Blood Pressure: MMDA Traffic Enforcers’ Health Study

**DOI:** 10.3390/ijerph182212122

**Published:** 2021-11-18

**Authors:** Zypher Jude G. Regencia, Godofreda V. Dalmacion, Antonio D. Ligsay, Emmanuel S. Baja

**Affiliations:** 1 Department of Clinical Epidemiology, College of Medicine, University of the Philippines Manila, Manila 1000, Philippines; zgregencia@up.edu.ph (Z.J.G.R.); gvdalmacion1@up.edu.ph (G.V.D.); 2 Institute of Clinical Epidemiology, National Institutes of Health, University of the Philippines Manila, Manila 1000, Philippines; 3 Professional Regulation Commission, Manila 1008, Philippines; 4 The Graduate School & College of Science, University of Santo Tomas, Manila 1008, Philippines; adligsay@ust.edu.ph; 5 Clinical Research Section, St. Luke’s College of Medicine-William H. Quasha Memorial, Quezon City 1112, Philippines

**Keywords:** black carbon, diastolic blood pressure, MMDA traffic enforcers, obesity, sex, systolic blood pressure

## Abstract

Exposure to traffic-related air pollution is linked with acute alterations in blood pressure (BP). We examined the cumulative short-term effect of black carbon (BC) exposure on systolic (SBP) and diastolic (DBP) BP and assessed effect modification by participant characteristics. SBP and DBP were repeatedly measured on 152 traffic enforcers. Using a linear mixed-effects model with random intercepts, quadratic (QCDL) and cubic (CCDL) constrained distributed lag models were fitted to estimate the cumulative effect of BC concentration on SBP and DBP during the 10 hours (daily exposure) and 7 days (weekly exposure) before the BP measurement. Ambient BC was related to increased BP with QCDL models. An interquartile range change in BC cumulative during the 7 days before the BP measurement was associated with increased BP (1.2% change in mean SBP, 95% confidence interval (CI), 0.1 to 2.3; and 0.5% change in mean DBP, 95% CI, −0.8 to 1.7). Moreover, the association between the 10-h cumulative BC exposure and SBP was stronger for female (4.0% change, 95% CI: 2.1–5.9) versus male and for obese (2.9% change, 95% CI: 1.0–4.8) vs. non-obese traffic enforcers. Short-term cumulative exposure to ambient traffic-related BC could bring about cardiovascular diseases through mechanisms involving increased BP.

## 1. Introduction

It is estimated that about 4.2 million people yearly across the world die from air pollution, according to the World Health Organization (WHO), and over 90% of people breathe air containing high levels of pollutants [1,2]. Exposure reduction to air pollution has an essential impact on global public health since the adverse health effects of air pollution are usually driven through their detrimental impact on cardiovascular health [3]. Counties are now confronting significant public health and climate crises brought about by air pollution, which prompted the WHO to update the existing guidelines on air pollution in September 2021. WHO provided more robust evidence to demonstrate how air pollution affects different aspects of health at even lower concentrations than previously known [4].

On average, an adult inhales 10–15 m^3^ of air per day, and it is inevitable to be exposed to various airborne pollutants [5]. The ambient air pollution components are classified into three main groups: (1) gases (e.g., NOx, CO, and O_3_); (2) volatile organic compounds (VOCs); and (3) suspended solid and liquid particles that are called particulate matters (PMs) [6,7]. Black carbon (BC) is a measured component of fine particulate matter (PM_2.5_) in the air [8]. BC usually exists in submicron particles from combustion-related sources, including transportation, fossil fuel burning, residential heating, and industry [9].

Relative to the other regions across the globe, Southeast Asia, where the Philippines is located, has one of the highest death ratios due to outdoor and household air pollution exposure [10,11,12]. In addition, lung cancer is the leading cause of cancer-related mortality in Southeast Asian men [13]. The problem is more severe in poor and developing countries due to overpopulation and uncontrolled urbanization, which leads to poor air quality, especially in areas with social disparities and poor management of the environment [14]. Numerous previous researches have evaluated the impact of pollution on health, which all concluded deleterious effects [15,16,17,18,19,20]. Moreover, chronic exposure is related to the detrimental impacts on cardiovascular health [21,22].

Several epidemiological studies have provided evidence that BC exposure is linked to detrimental health effects [8,23,24,25]. It was emphasized by various researches the potential role of BC in influencing the variability of toxicologically active components of PM_2.5_ [26,27]. BC in urban environments is often related to adverse respiratory and cardiovascular effects, including an increase in blood pressure (BP) [28], increased cases of asthma, and premature deaths [29]. Unsustainable urbanization and outdated environmental protection legislation, which resulted in severe degradation of urban air quality in terms of BC emission, exacerbate these problems in developing megacities in Southeast Asia, Latin America, and Africa [30]. There remain regional differences in BC-related health effects described in some epidemiological researches that cannot be fully explained by geographical variations in ambient concentrations of BC [9].

Several studies concluded that exposure to BC is associated with cardiovascular effects [9,31,32]. Although the evidence linking BC to subclinical cardiovascular endpoints is more limited than that for PM_2.5_, BC is of interest from a health perspective because multiple studies report associations between combustion-related air pollution and health effects [31,33,34,35]. In addition, acute and chronic BC exposures contribute to cardiovascular morbidity and mortality [36,37]. Physiological events happen to mediate these cardiovascular effects, such as an altered autonomic function of the heart, changes in micro- and macrovascular reactivity, induction of systemic inflammation, endothelial dysfunction, and altered peripheral resistance of the blood vessels [38,39,40,41]. Moreover, elevation in BP and an increased risk for developing hypertension usually result from microcirculation, which determines the overall peripheral resistance and microvascular alterations [28]. One study in Beijing, China, demonstrated the impact of short-term variation in high levels of ambient air pollution metrics on health outcomes. Results showed that both PM_2.5_ and BC are linked to BP increase and insulin resistance, even among at-risk individuals who have been living for long periods in these conditions [42]. The study also demonstrated that personal exposure to BC elevates blood pressure and heart rate within a few hours [42].

In less developed and developing countries, megacities endure high levels of traffic-related air pollution and its health effects due to rapid urbanization, modernization, and economic growth [29]. Metro Manila, Philippines, is one of those megacities and home to approximately 13 million people. The air quality situation in Metro Manila is frequently hazardous due to various sources of pollution, and the residents intermingle in condensed, limited spaces [30]. Furthermore, Metro Manila is reported to have the highest number of registered motor vehicles compared with other cities in the Philippines. Based on the National Emissions Inventory by source conducted in 2015, the majority (65%) of air pollutants in the Philippines came from mobile sources, such as cars, motorcycles, diesel trucks, and buses. Moreover, almost 21% were contributed by stationary sources, such as power plants and factories, and the rest (14%) were from area sources, such as construction activities and open burning of solid wastes. Conversely, Metro Manila inventory in the same year revealed that mobile sources contributed 88% to total air pollution in the area compared to 10% from stationary sources and a mere 2% from area sources [43].

There is a need to understand better the association between air pollution and health outcomes, including BC and BP levels. Our study examined the association between acute cumulative exposure to BC and BP, particularly systolic (SBP) and diastolic (DBP) BP, among urban traffic enforcers stationed along a major circumferential highway in Metro Manila. Moreover, we evaluated effect modification by the characteristics of the traffic enforcers.

## 2. Materials and Methods

### 2.1. Study Population and Study Site

The study population involved 152 traffic enforcers who were actively employed and working for the Metropolitan Manila Development Authority (MMDA). Traffic enforcers stationed along the Epifanio de Los Santos Avenue (EDSA) were randomly selected from the list given by MMDA management. Recruitment was voluntary on the traffic enforcers and included 19–65-year-old traffic enforcers working the 5:00 am to 2:00 pm shift from May 2014 to April 2015. In addition, traffic enforcers working on secondary roads and other highways, pregnant, and clinically diagnosed with pulmonary tuberculosis were excluded from the study. The study received ethics approval from an independent Ethics Review Committee.

### 2.2. Data Collection

#### 2.2.1. Health Assessment and Self-Administered Questionnaire

As part of the baseline health assessment, weight and height measurements and collection of serum samples were done on all eligible traffic enforcers, including a self-administered questionnaire containing data on medical history, medication usage, alcohol consumption, smoking history, and other factors that could affect health. These measurements were done the day before the start of the workweek of the traffic enforcers. Furthermore, the traffic enforcers were asked to report four more visits to the MMDA command center clinic for additional BP measurements and blood extractions after their daily duty.

#### 2.2.2. Blood Pressure Measurement

For the health outcome assessment, sitting SBP and DBP measurements of the traffic enforcers were recorded, and OMRON^®^ BP monitor (manufactured by OMRON Asia Pacific Pte Ltd., Singapore 119967) was used as an instrument (BP reading: within ± 3.0 mmHg or 2 percent, conforming with the Association of Medical Instrumentation (AAMI) standards). The research team assessed the SBP and DBP of all the traffic enforcers at the end of their scheduled duty (between 2 pm to 3 pm) at the MMDA command center clinic. Traffic enforcers were seated for 5 min with arms at the level of the heart before the SBP and DBP measurements. The average of three readings of SBP and DBP was used in the analysis. If the traffic enforcer was agitated, upset, or excited, an additional 10–15 min of sitting before the measurement of SBP and DBP was done. In addition, the participants were advised to avoid food, alcohol, caffeine, and tobacco for 30–60 min before measuring their SBP and DBP.

#### 2.2.3. Black Carbon and Meteorological Measurement

Ambient BC levels were measured daily using a real-time, pocket-sized BC aerosol monitor (microAeth^®^ Model AE51, manufactured by AethLabs, Inc., San Francisco, CA 94110, USA). The monitor operated continuously for 24 h and was positioned on the rooftop of the Metro Base Ver. 2.0 MMDA station command center at Orense Guadalupe, Makati, Philippines. For quality assurance of BC measurements, a second BC aerosol monitor was co-located at the rooftop site. Relative humidity (RH) and ambient temperature (AT) measurements were obtained from the Philippine Atmospheric, Geophysical, and Astronomical Services Administration—Department of Science and Technology (PAGASA–DOST) weather stations located in Science Garden, Quezon City. Moreover, the study had 570 valid BC exposure and BP outcome measurements of the 152 traffic enforcers who had one (*n* = 15), two (*n* = 23), three (*n* = 25), four (*n* = 11), or five (*n* = 78) measurements.

### 2.3. Data Analysis

Descriptive statistics and correlation coefficients were calculated to describe and evaluate the relationships among BC, SBP, DBP, and covariates. Associations between BC and the change in the mean of both systolic BP (SBP) and diastolic BP (DBP) were estimated using linear mixed-effects with random subject-specific intercepts regression models with an unstructured covariate matrix structure. It is a standard approach required to capture and account for the residual correlation among measurements within the same traffic enforcer and to take into account the heterogeneity in the traffic enforcer’s overall BP measurements for the repeated measures data [44,45].

Two BC-pollutant models were assessed. First, to examine the workday and 1-week exposure windows of traffic enforcers to BC and to minimize multiple comparisons, the study fitted quadratic constrained distributed lag (QCDL), cubic constrained distributed lag (CCDL), and BC-pollutant models to estimate the cumulative effect of BC during a 10-h and a 7-day time window before the visit for the measurement of blood pressure [46]. The linear mixed-effects regression models for the cumulative effect of BC on BP take the general equation:

Quadratic Constrained Distributed Lag Linear Mixed-Effects Model (QCDL):

(1) $$E\left(Y_{ij}\right)=b_{0i}+\beta_{0}+\alpha_{0}\left(\overset{q}{\underset{k=0}{\sum }}k^{0}BC_{ij-k}\right)+\alpha_{1}\left(\overset{q}{\underset{k=0}{\sum }}k^{1}BC_{ij-k}\right)+\alpha_{2}\left(\overset{q}{\underset{k=0}{\sum }}k^{2}BC_{ij-k}\right)+f\left(s_{ij}\right)+X_{ij}^{T}\beta$$

(2) $$E\left(Y_{ij}\right)=b_{0i}+\beta_{0}+\alpha_{0}v_{0ij}+\alpha_{1}v_{1ij}+\alpha_{2}v_{2ij}+f\left(s_{ij}\right)+X_{ij}^{T}\beta$$

Cubic Constrained Distributed Lag Linear Mixed-Effects Model (CCDL):

(3) $$E\left(Y_{ij}\right)=b_{0i}+\beta_{0}+\alpha_{0}\left(\overset{q}{\underset{k=0}{\sum }}k^{0}BC_{ij-k}\right)+\alpha_{1}\left(\overset{q}{\underset{k=0}{\sum }}k^{1}BC_{ij-k}\right)+\alpha_{2}\left(\overset{q}{\underset{k=0}{\sum }}k^{2}BC_{ij-k}\right)+\alpha_{3}\left(\overset{q}{\underset{k=0}{\sum }}k^{3}BC_{ij-k}\right)+f\left(s_{ij}\right)+X_{ij}^{T}\beta$$

(4) $$E\left(Y_{ij}\right)=b_{0i}+\beta_{0}+\alpha_{0}v_{0ij}+\alpha_{1}v_{1ij}+\alpha_{2}v_{2ij}+\alpha_{3}v_{3ij}+f\left(s_{ij}\right)+X_{ij}^{T}\beta$$

where *Y_ij_* is the BP (SBP or DBP) measurement of traffic enforcer *i* at time *j*, *β_0_* is the overall intercept, *b_0i_* is the random intercept for traffic enforcer *i*, *ν_mij_* is a linear combination of the current and lagged values of BC pollution *BC_ij_* (q = 1 to 10 h, or q = 1 to 7 days), *f(s_ij_)* is the smooth function of calendar date (natural spline with 4 degrees of freedom), and *X*_1*ij*_,…, *X_rij_* are the parametric fixed effects covariate terms from 1 to r identified *a priori* and measured at each visit in which the SBP and DBP measurements were taken. Based on the established relationship between BP and BC [47], the following covariates were controlled *a priori* in all the models: age, sex, body mass index (BMI), fasting blood glucose level, cholesterol level, hypertensive status (yes, no), cigarette smoking status (never, ever), alcohol drinking status (never, ever), and years of work experience [48,49,50]. In addition, we included the graduate of a 4-year degree (Yes/No) variable in the model to control for the socioeconomic status. Furthermore, we used a natural spline of calendar date with 4 degrees of freedom in the model to account for the variation in season and other possible long-term time trends in blood pressure. Moreover, we also modeled traffic enforcer’s duty-post effects with indicator variables and controlled 1-h mean relative humidity and temperature using linear terms. The Akaike Information Criterion (AIC) was used to determine the best model fit for the cumulative effect of BC on BP, with QCDL giving a better model fit than CCDL for both exposure windows (e.g., AIC for 10-h exposure window cumulative effect on DBP: QCDL −715.4.0 vs. CCDL −698.3; AIC for 7-d exposure window cumulative effect on SBP: QCDL −880.5 vs. CCDL −864.0).

For effect modification by participant characteristics, traffic enforcers were classified into two groups according to the following characteristics: sex (male; female), obesity status (obese, BMI ≥ 30 kg/m^2^; non-obese, BMI < 30 kg/m^2^), smoking status (ever smoker, never smoker), and drinking status (ever drinker, never drinker). Interaction terms between BC and the dichotomized effect modifier were included in the models. We reported the effect size estimates with a 95% confidence interval (95% CI) as percent change in mean BP (SBP or DBP) per interquartile range (IQR) change of BC. R Studio (Version 1.1.463—© 2021—2018 RStudio, Inc., Boston, MA, USA) was used in the analyses.

## 3. Results

### 3.1. Demographic Profile and Health Assessment

Five hundred and seventy valid BP and BC measurements available for analysis were collected from 152 eligible MMDA traffic enforcer participants. The traffic enforcers were middle-aged men with a mean age (±SD) of 37.2 years ± 8.7 years who were generally slightly overweight with a mean BMI (±SD) of 25.9 ± 4.2 kg/m^2^. In addition, approximately three out of four traffic enforcers were drinkers, and 3 out of 20 were hypertensive. The mean SBP of the traffic enforcers was slightly elevated (128.2 mmHg ± 16.2 SD) than the standard average SBP of 120.0 mmHg, while the mean DBP of the traffic enforcers was less than (78.1 mmHg ± 11.1 SD) the acceptable normal DBP of 80 mmHg. Table 1 shows the other characteristics of the study participants. 

Table 2 summarizes the exposure and meteorological variable measurements, including descriptive statistics of ambient BC, AT, and RH measurements. Ambient BC mean concentrations during the current measurement (0-h lag), 10-h lag, and 7-day lag prior to health measurements were 10.6 μg/m^3^ ± 10.4 SD, 8.1 μg/m^3^ ± 5.1 SD, and 9.3 μg/m^3^ ± 8.6 SD, respectively.

### 3.2. Effect of Black Carbon on Blood Pressure

**Figure 1:**
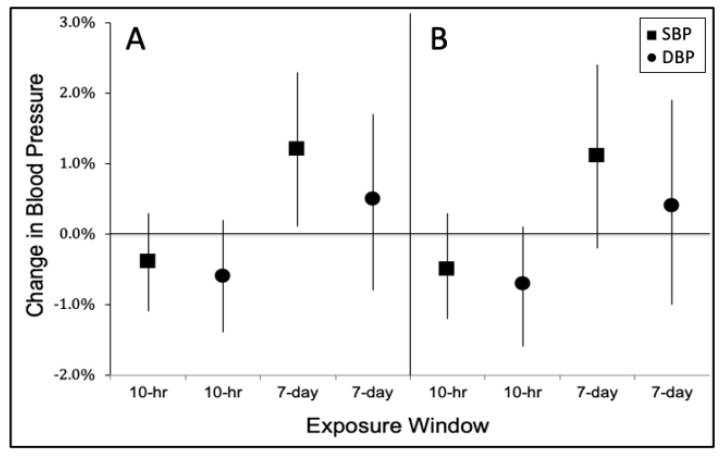
Adjusted effect estimates of percent change in systolic (SBP) and diastolic (DBP) blood pressure per interquartile range (IQR) change in cumulative exposure to black carbon during the 10 h and 7 days before SBP and DBP measurements: pollutant model with random intercept using quadratic constrained distributed lags (**A**) and cubic constrained distributed lags (**B**). All models were adjusted for age, sex, body mass index, fasting blood glucose level, cholesterol level, hypertension status (yes, no), cigarette smoking status (never, ever), alcohol drinking status (never, ever), traffic enforcer’s duty post, work experience, graduate of a 4-year degree (yes, no), temperature, relative humidity, and a natural spline for long-term time trend (date). Error bars indicate 95% Confidence Interval.

Figure 1 compares the estimates for the cumulative BC exposure for both the 10-h and the 7-day time window lag models. The results from both QCDL and CCDL models produced almost similar adjusted effect estimates and 95% confidence intervals, with QCDL models exhibiting better Akaike Information Criterion model fit than the CCDL models. In the QCDL model, ambient BC was positively associated with both SBP and DBP for the 7-day cumulative exposure before the BP measurement (SBP: 1.2 percent change in mean SBP per IQR increase in cumulative BC exposure; 95% confidence interval (CI), 0.1 to 2.3; DBP: 0.5% change in mean DBP per IQR increase in cumulative BC exposure; 95% CI, −0.8 to 1.7;). In contrast, for the 10-h cumulative exposure, we found null associations between ambient BC and blood pressure (SBP: −0.4 % change in mean SBP per IQR increase in cumulative BC exposure; 95% CI, −1.1 to 0.3; DBP: −0.6 % change in mean DBP per IQR increase in cumulative BC exposure; 95% CI, −1.4 to 0.2, see Table S1 for more details).

**Figure 2:**
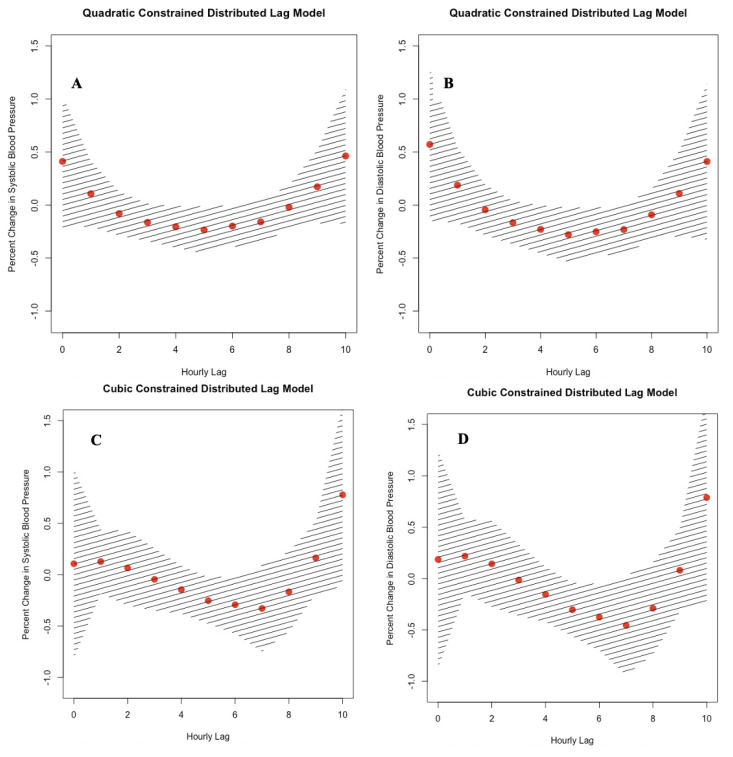

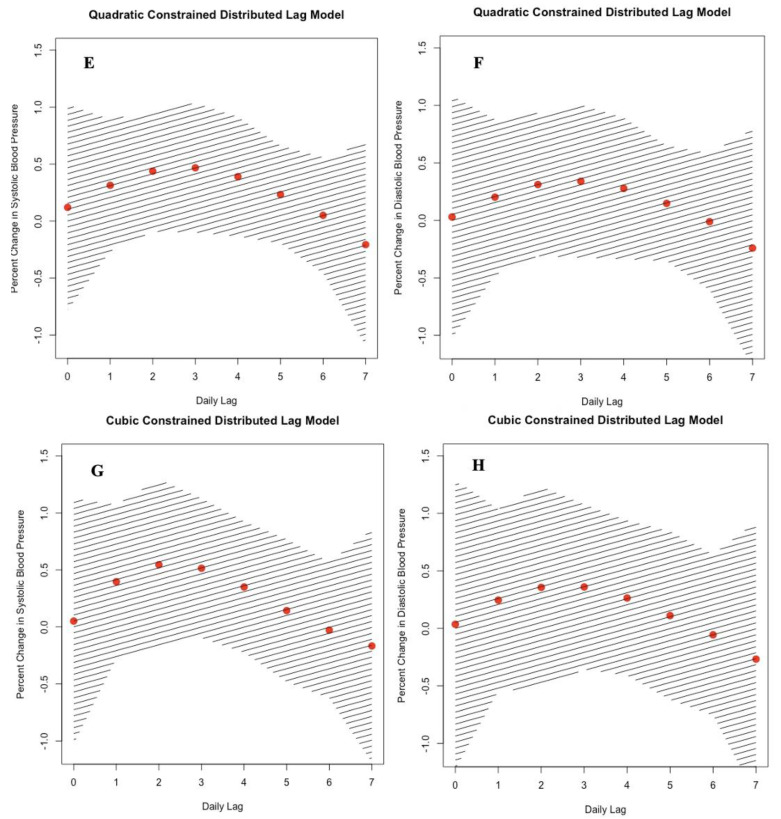
Adjusted effect estimates of percent change in systolic (SBP) and diastolic (DBP) blood pressure per interquartile range (IQR) change of traffic-related black carbon. (**A**–**D**) Pollutant model of cumulative exposure for a 10-h time window with random intercept using quadratic constrained distributed lag (QCDL) with hourly lags [SBP (**A**), and DBP (**B**)] and cubic constrained distributed lag (CCDL) with hourly lags [SBP (**C**), and DBP (**D**)]. Adjusted effect estimates of percent change in SBP and DBP blood pressure per IQR change of traffic-related black carbon. (**E**–**H**) Pollutant model of cumulative exposure for a 7-day time window with random intercept using QCDL with daily lags [SBP (**E**), and DBP (**F**)] and CCDL with daily lags [SBP (**G**), and DBP (**H**)]. Hatch-marked regions indicate 95% Confidence Interval.

Figure 2 compares the pollutant model plots for the 10-h and 7-day cumulative BC exposure time windows using QCDL and CCDL with hourly and daily lags. In addition, we found wider confidence intervals in the 7-day exposure time window as compared with the 10-h exposure time window for both the QCDL and CCDL models.

### 3.3. Effect Modification by Participant Characteristics

**Figure 3:**
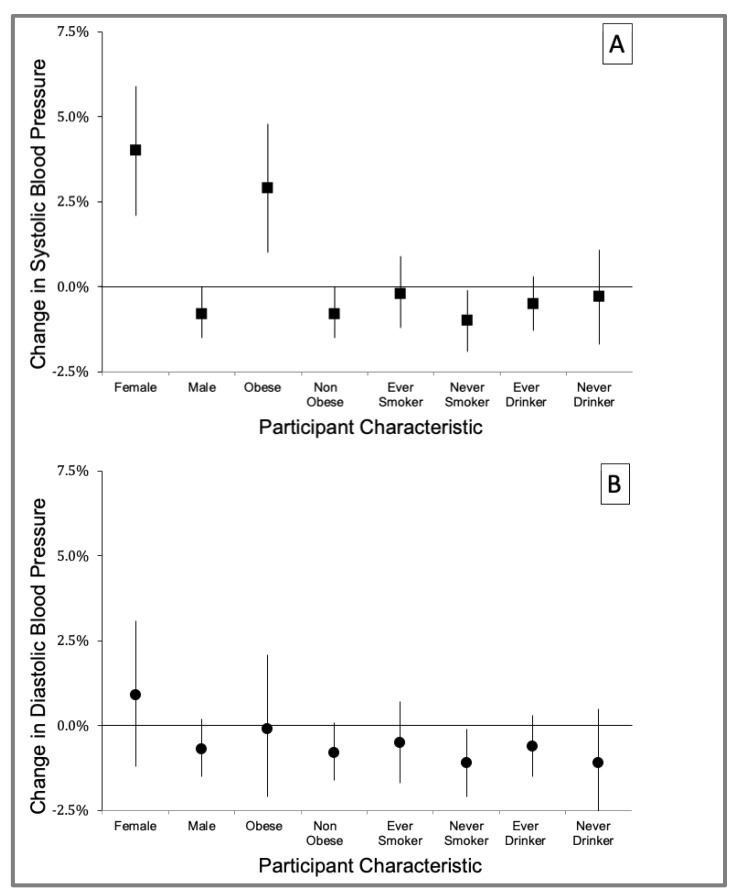
Adjusted change in mean systolic (**A**) and diastolic (**B**) blood pressure per IQR change of traffic-related black carbon: cumulative exposure during 10 h before blood pressure measurement, by participant characteristics [sex (male, female), obesity status (obese: body mass index ≥ 30, non-obese: body mass index < 30), smoking status (ever smoker, never smoker), and drinking status (ever drinker, never drinker)]. Error bars indicate 95% Confidence Interval.

We assessed whether being a drinker, obese, smoker, or male or female modified the effect of BC on SBP and DBP. Figure 3 shows the results of effect modification by participant characteristics for the QCDL model for the cumulative BC exposure during the 10 h before BP measurement. BC was positively associated with SBP with a 4.0% (95% CI: 2.1 to 5.9) change in mean SBP among female traffic enforcers but was negatively associated (−0.8% change; 95% CI: −1.5 to 0.0) for male traffic enforcers. In addition, the association between BC and SBP was also stronger for obese (2.9% change, 95% CI: 1.0 to 4.8) versus non-obese (−0.8% change, 95% CI: −1.5 to 0.0) traffic enforcers. No effect modification by smoking and drinking was observed for the effect of BC on both SBP and DBP (see Table S2 for more details).

**Figure 4:**
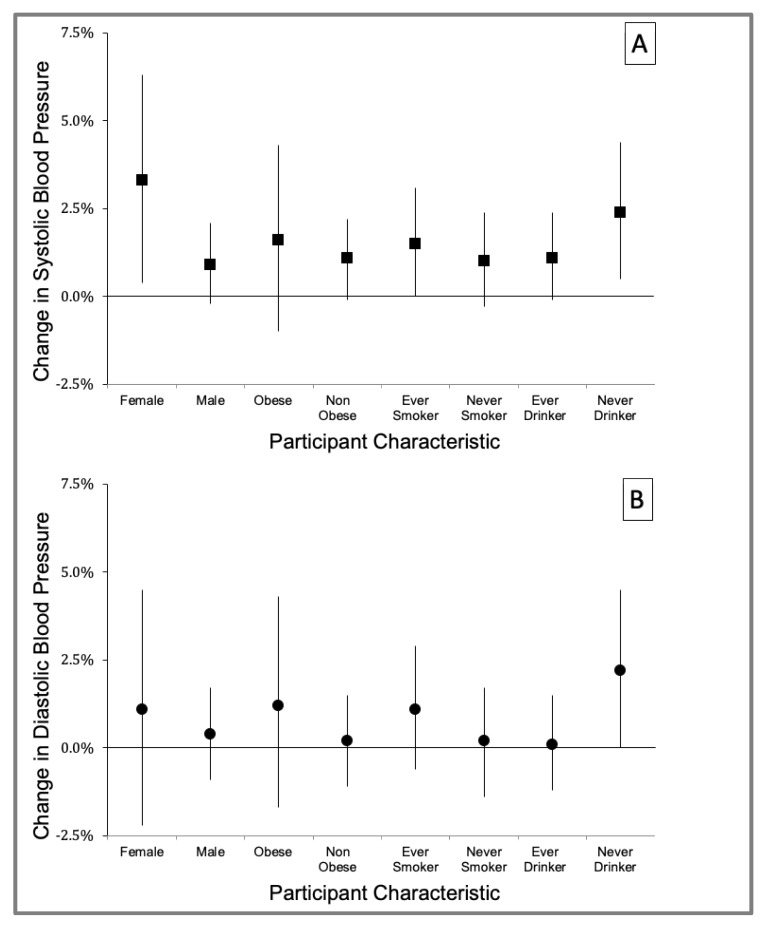
Adjusted change in mean systolic (**A**) and diastolic (**B**) blood pressure per IQR change of traffic-related black carbon: cumulative exposure during 7 days before blood pressure measurement, by participant characteristics (sex (male, female), obesity status (obese: body mass index ≥ 30, non-obese: body mass index < 30), smoking status (ever smoker, never smoker), and drinking status (ever drinker, never drinker)). Error bars indicate 95% Confidence Interval.

Figure 4 shows the results of effect modification by sex, obesity, drinking, and smoking status for the cumulative BC exposure during the 7 days before BP measurement using QCDL models. The association between the BC and SBP was more robust among female participants (3.3% change, 95% CI: 0.4 to 6.3) than male participants (0.9% change, 95% CI: −0.2 to 2.1) and never drinking (2.4% change, 95% 0.5 to 4.4) versus ever drinking (1.1% change, 95% CI: −0.1 to 2.4) traffic enforcers (Table S3 for more details). 

## 4. Discussion

Our study provides a significant suggestion that acute cumulative exposure for seven days to traffic-related BC is associated with increased SBP, a risk factor for stroke and heart attack. In contrast, null associations were found between BC and both SBP and DBP for a 10-h cumulative exposure window. Moreover, results from our study also suggest that female, and obese traffic enforcers remain predominantly at risk from traffic-related BC exposure.

As a by-product of the incomplete combustion of fossil fuels, BC is among the prevalent ambient particles in the world. Exposure to traffic-related BC has a well-documented association with numerous adverse health outcomes, and the association of BC with increased systolic BP is among the most extensively studied [51,52,53]. Research reported that the emission in Manila is dominated by ultrafine particles comprising about 90% of the total ambient air pollution. In addition, BC emission shared up to 70% of the calculated emission factors of particle number. This finding implies that the urban air in Manila comprises high concentrations of ultrafine particles and BC particles, which are highly toxic [54]. EDSA is considered the busiest highway in Metro Manila. According to MMDA data in 2013, 156,000 vehicles, including diesel buses, provincial and local, represent a large part of EDSA traffic. This observation results in low profitability, excessive BC air pollution levels and mediocre consideration for passengers [55]. Furthermore, our findings suggest an acute 1-week cumulative effect of ambient BC exposure on SBP. The strong association between traffic enforcers’ BP and ambient BC implies that ambient BC effects are linked to background levels of traffic-related BC pollution in Metro Manila, particularly to traffic enforcers’ occupational health-related exposure.

Our findings were consistently in agreement with previous studies that also examined the acute exposure effect of BC on BP [38,42,50,56,57,58,59,60,61,62]. For example, the 7-day cumulative exposure to ambient BC and its positive effect on SBP and DBP findings from our study of traffic enforcers were supported by those from an aging cohort study of older men [38,50], a cohort of participants with metabolic syndrome [42], a panel study of subjects with Type 2 diabetes mellitus [58], and a study on hypertensive patients [62]. In addition, our results of null associations between a 10-h cumulative exposure window to ambient BC and both SBP and DBP were comparable to a cross-over study of women [57] and a cohort of community dwellers living near major highways [56]. These studies showed null effects in SBP and DBP for a 3-h [57] and 24-h [56] prior exposure windows. However, some studies did not concur with our findings, including a controlled cross-over study of healthy nonsmoking adults [59] and a non-smoking elderly cohort [61], with both studies reporting positive associations on SBP and DBP. We hypothesize that the differences may be due to a much higher BC exposure level in the cross-over study than in our study, which may significantly heighten the effect of BC on SBP. In addition, the elderly cohort study involved a vulnerable population that is more susceptible to the health effects of BC.

Elevated BP has been considered worldwide as the most substantial modifiable risk factor for cardiovascular disease and related disability [63]. In 2016, air pollution was the fifth leading risk factor for the global burden of disease, with ambient BC pollution as the most important cause of illness among environmental risk factors. It led to 4.0 million deaths and 105.7 million disability-adjusted life years [1]. Elevated BP levels or less vasodilatation were found in humans experimentally exposed to concentrated ambient air pollution [64]. Components of particles, especially ultrafine particles, induce oxidative stress responses [65], an important mechanism underlying hypertension [66].

Analysis of the effect modification by sex in our study indicated that the associations between the cumulative ambient BC exposure windows of 10 h and 7 days and elevated SBP were more robust for female than male traffic enforcers. Our findings did not agree with a cross-sectional study of police officers that reported men to have a significantly higher SBP than women in pre- and post-shift. The SBP of women was considerably higher post-shift than pre-shift [67]. This difference may be attributed to the higher sample size of female participants of the cross-sectional study compared to our study. Furthermore, male participants predominately have higher SBP than female participants in developing populations [68]. However, women may be more susceptible than men to the particulate matter-induced health effects; robust risk estimates have been reported for studies that found an increase in fatal coronary heart disease and cardiovascular events [69,70,71]. Our observed effect modification by sex needs to be verified in future studies.

Evidence of effect modification by obesity was observed on the association between 10 h before BP measurement cumulative BC exposure and elevated SBP. In one study, obesity and blood pressure are associated; an increase in BMI is related to an increase in systolic blood pressure [72]. This study may support our finding of a stronger association between ambient BC and SBP among obese compared with non-obese traffic enforcers. Nonetheless, our results suggest that traffic enforcers who are female or obese may be more responsive to traffic-related ambient BC than traffic enforcers who are male or not obese.

Several studies have delved into exposure assessment and characterization of particulate matter, including black carbon in Metro Manila; however, the health effects of ambient black carbon exposure on susceptible populations in Metro Manila were never examined [30,54,73]. Similar to our health effects study of airborne heavy metals on BP [74], this study is the first study that looked at the association between acute ambient BC exposure and BP in the Philippines. Using a linear mixed model with random subject-specific intercept means that contrasts were both within and between subjects; therefore, bias due to confounding factors should be less than that observed in cross-sectional study design [45]. However, bias as a result of unmeasured or residual confounding can never be disregarded. In addition, caution must be made in generalizing that female traffic enforcers are more susceptible to an increase in SBP than male traffic enforcers because of the small sample size of female participants included in our study. Future research to validate this finding must be conducted.

Another limitation of our study is the BC exposure monitoring. We used a single site as a surrogate for acute ambient black carbon exposure, which will not pick up the spatial variation in BC concentrations. With the traffic enforcers stationed ~5 km on a median straight-line distance from our ambient monitoring site, adequate spatial variation in BC concentrations may be observed. Consequently, this spatial variation may lead to exposure misclassification that would likely be nondifferential and could bias our estimates toward the null [75]. Furthermore, our study utilized Filipino traffic enforcers who were young and predominantly healthy as the study population. Thus, the results of this study may not be generalizable to older people, children, or other ethnic and racial groups. Future studies on the health effect of ambient BC on BP on these at-risk populations should be considered.

## 5. Conclusions

Our study supports the health effects of elevated short-term cumulative exposure to ambient BC on increased SBP, a cardiovascular risk marker among urban traffic enforcers in Metro Manila, Philippines. In addition, male traffic enforcers appear to be better protected from SBP elevation with ambient BC exposure than female traffic enforcers. Moreover, when cumulatively exposed to ambient BC, even in shorter exposure windows, non-obese traffic enforcers tend to be protected against increased SBP compared with obese traffic enforcers. The findings of our study further suggest that short-term cumulative exposure to ambient BC could bring about cardiovascular diseases through mechanisms involving increased BP.

## Figures and Tables

**Table 1:** Characteristics of the study population (*N* = 152).

Characteristics	Mean ± SD, %
Age, years	37.2 ± 8.7
Male	90.1
Cholesterol level, mg/dL	203.5 ± 38.4
Glucose fasting, mg/dL	102.6 ± 47.5
Body mass index, kg/m^2^	25.9 ± 4.2
Obese (BMI ≥ 30 kg/m^2^)	16.4
Hypertensive	15.8
Ever cigarette smoker	44.1
Ever drinker	76.3
College graduate	36.2
Work experience, years	9.7 ± 8.6
Systolic blood pressure, mmHg	128.2 ± 16.2
Diastolic blood pressure, mmHg	78.1 ± 11.1

**Table 2:** Concentration of temperature, relative humidity, and black carbon during (0-h), 10-h, or 7-days before blood pressure monitoring.

Variable	Lag	Mean ± SD	Median	IQR
Black carbon, μg/m^3^	0-h	10.6 ± 10.4	6.1	10.8
	10-h	8.1 ± 5.1	7.3	7.1
	7-d	9.3 ± 8.6	5.8	8.5
Temperature, °C ^a^		32.0 ± 2.6	32.1	3.4
Relative humidity, % ^b^		55.8 ± 11.7	54.4	15.7

^a^ Current 1-h mean temperature; ^b^ Current 1-h mean relative humidity.

## Data Availability

The data are not publicly available due to privacy or ethical restrictions.

## Supplementary Materials

The following are available online at https://www.mdpi.com/article/10.3390/ijerph182212122/s1: Table S1: Adjusted effect estimates of percent change in systolic (SBP) and diastolic (DBP) blood pressure per interquartile range (IQR) change in cumulative exposure to black carbon during the 10 h and 7 days before SBP and DBP measurements: pollutant model with random intercept using QCDL or CCDL. All models were adjusted for age, sex, BMI, cholesterol, hypertensive (yes, no), cigarette smoker (never, ever), alcohol drinker (yes, no), fasting blood glucose (FBG) level, years of service, graduate of a 4-year degree (yes, no), traffic enforcer’s duty post, temperature, relative humidity, and a natural spline for long–term time trend (date, degrees of freedom = 4); Table S2: Adjusted effect estimates of percent change in systolic (SBP) and diastolic (DBP) blood pressure per interquartile range (IQR) change in cumulative exposure to black carbon during the 10 h before SBP and DBP measurements, by participant characteristics [sex (male, female), smoking (ever, never), obesity (obese (+): body mass index ≥ 30, non-obese (−): body mass index < 30), smoking status (ever smoker, never smoker), and drinking status (drinker (+): yes, drinker (−): no)]; and Table S3: Adjusted effect estimates of percent change in systolic (SBP) and diastolic (DBP) blood pressure per interquartile range (IQR) change in cumulative exposure to black carbon during the 7 days before SBP and DBP measurements, by participant characteristics [sex (male, female), smoking (ever, never), obesity (obese (+): body mass index ≥ 30, obese (−): body mass index < 30), smoking status (ever smoker, never smoker), and drinking status (drinker (+): yes, drinker (−): no)].
