# Supplementary material for: Short-Term Cumulative Exposure to Ambient Traffic-Related Black Carbon and Blood Pressure: MMDA Traffic Enforcers’ Health Study
Source: Int J Environ Res Public Health. 2021 Nov 18;18(22):12122. doi: 10.3390/ijerph182212122 (PMC8619089; doi:10.3390/ijerph182212122)
Supplement: Supplementary file 1 [file ijerph-18-12122-s001.zip › ijerph-1420194-supplementary.pdf]

Table S1. Adjusted effect estimates of percent change in systolic (SBP) and diastolic (DBP) blood pressure per interquartile range (IQR) change in cumulative exposure to black carbon during the 10 hours and 7 days before SBP and DBP measurements: pollutant model with random intercept using QCDL or CCDL. All models were adjusted for age; sex; BMI; cholesterol; hypertensive (yes, no); cigarette smoker (never, ever); alcohol drinker (yes, no); fasting blood glucose (FBG) level; years of service; graduate of a 4-year degree (yes, no); traffic enforcer's duty post; temperature; relative humidity; and a natural spline for long–term time trend (date, degrees of freedom = 4).

Health Outcome/ Exposure Window	Quadratic Constrained Distributed Lag	Cubic Constrained Distributed Lag
	% Change in Blood Pressure (95% Confidence Interval)	
**Systolic Blood Pressure**		
10 hours	-0.4 (-1.1, 0.3)	-0.5 (-1.2, 0.3)
7 days	1.2 (0.1, 2.3)	1.1 (-0.2, 2.4)
**Diastolic Blood Pressure**		
10 hours	-0.6 (-1.4, 0.2)	-0.7 (-1.6, 0.1)
7 days	0.5 (-0.8, 1.7)	0.4 (-1.0, 1.9)

Table S2. Adjusted effect estimates of percent change in systolic (SBP) and diastolic (DBP) blood pressure per interquartile range (IQR) change in cumulative exposure to black carbon during the 10 hours before SBP and DBP measurements, by participant characteristics [sex (male, female), smoking (ever, never), obesity (obese (+): body mass index ≥ 30, obese (-): body mass index < 30), smoking status (ever smoker, never smoker), drinking status (drinker (+): yes, drinker (-): no), and hypertension status (hyper (+): yes, hyper (-): no)].

Parameters/Health Outcome	Modifiers	% Change in Blood Pressure (95% Confidence Interval)
Sex		
Systolic blood pressure	Female	4.0 (2.1, 5.9)
	Male	-0.8 (-1.5, 0.0)
Diastolic blood pressure	Female	0.9 (-1.2, 3.1)
	Male	-0.7 (-1.5, 0.2)
Obesity Status		
Systolic blood pressure	Obese	2.9 (1.0, 4.8)
	Non-obese	-0.8 (-1.5, 0.0)
Diastolic blood pressure	Obese	-0.1 (-2.1, 2.1)

	Non-obese	-0.8 (-1.6, 0.1)
Smoking status		
Systolic blood pressure	Ever	-0.2 (-1.2, 0.9)
	Never	-1.0 (-1.9, -0.1)
Diastolic blood pressure	Ever	-0.5 (-1.7, 0.7)
	Never	-1.1 (-2.1, -0.1)
Drinking status		
Systolic blood pressure	Drinker	-0.5 (-1.3, 0.3)
	Non-drinker	-0.3 (-1.7, 1.1)
Diastolic blood pressure	Drinker	-0.6 (-1.5, 0.3)
	Non-drinker	-1.1 (-2.6, 0.5)

Table S3. Adjusted effect estimates of percent change in systolic (SBP) and diastolic (DBP) blood pressure per interquartile range (IQR) change in cumulative exposure to black carbon during the 7 days before SBP and DBP measurements, by participant characteristics [sex (male, female), smoking (ever, never), obesity (obese (+): body mass index ≥ 30, obese (-): body mass index < 30), smoking status (ever smoker, never smoker), drinking status (drinker (+): yes, drinker (-): no), and hypertension status (hyper (+): yes, hyper (-): no)].

Parameters	Modifiers	% Change in Blood Pressure (95% Confidence Interval)

Sex		
Systolic blood pressure	Male	0.9 (-0.2, 2.1)
	Female	3.3 (0.4, 6.3)
Diastolic blood pressure	Male	0.4 (-0.9, 1.7)
	Female	1.1 (-2.2, 4.5)
Obesity Status		
Systolic blood pressure	Obese	1.6 (-1.0, 4.3)
	Non-obese	1.1 (-0.1, 2.2)
Diastolic blood pressure	Obese	1.2 (-1.7, 4.3)
	Non-obese	0.2 (-1.1, 1.5)
Smoking status		
Systolic blood pressure	Ever	1.5 (0.0, 3.1)
	Never	1.0 (-0.3, 2.4)
Diastolic blood pressure	Ever	1.1 (-0.6, 2.9)
	Never	0.2 (-1.4, 1.7)
Drinking status		
Systolic blood pressure	Drinker	1.1 (-0.1, 2.4)
	Non-drinker	2.4 (0.5, 4.4)
Diastolic blood pressure	Drinker	0.1 (-1.2, 1.5)
	Non-drinker	2.2 (0.0, 4.5)
